# Replication stress: an early key event in ochratoxin a genotoxicity?

**DOI:** 10.1007/s00204-025-04004-4

**Published:** 2025-03-10

**Authors:** C. Klotz, J. Borchers, J. Brode, P. Lambeck, A. Mally

**Affiliations:** Department of Toxicology, University of Würzburg, Versbacher Str. 9, 97078 Würzburg, Germany

**Keywords:** Ochratoxin A, Food contaminant, Carcinogenesis, Genotoxicity, Replication stress, DNA replication, DNA damage, γH2AX

## Abstract

Ochratoxin A (OTA), a widespread food contaminant and potent renal carcinogen in rodents, is weakly genotoxic in mammalian cells. The mechanisms underlying OTA-induced genetic damage are still poorly understood. In its recent risk assessment, the European food safety authority (EFSA) considered that the specific spectrum of mutations and chromosomal damage induced by OTA may derive from unresolved replication stress. The aim of the present work was to experimentally test the hypothesis that OTA interferes with DNA replication and to characterize the cellular response to OTA-mediated replication stress. Using the DNA fiber assay to study replication fork dynamics at single molecule resolution, a small but statistically significant global delay in replication fork progression was observed in human kidney (HK-2) cells exposed to OTA at ≥ 10 µM. OTA-mediated interference with DNA replication was confirmed by a concentration-related decrease in incorporation of the thymidine analog 5-ethynyl-2′-deoxyuridine (EdU) into newly replicating DNA in HK-2 cells arrested in late G_1_/S via double thymidine block and treated with OTA during S phase. Western blot and immunofluorescence analyses revealed a significant concentration-related increase in γH2AX in cells exposed to OTA. Co-localization of γH2AX foci with 5-chloro-2′-deoxyuridine (CldU) incorporated into cells during S phase and increased γH2AX labeling along newly replicating chromatin fibers visualized using the extended chromatin fiber assay support a replication-coupled mechanism of OTA-induced DNA damage. Experiments with cells synchronized in late G_1_/S or late G_2_ demonstrated that exposure of cells to OTA during S phase, but not mitosis, leads to a significant concentration-related increase in H2AX, providing further evidence that OTA may act primarily during S phase of the cell cycle. However, OTA did not appear to efficiently activate ATR-Chk1 and ATM-Chk2 DNA damage response pathways, suggesting that cells with under-replicated DNA or unresolved DNA damage may escape checkpoint control and may continue into mitosis, with potentially deleterious consequences for genomic integrity. Overall, results from this study provide first experimental evidence for perturbation of the S phase replisome machinery by OTA and point toward replication stress as an early key event in OTA genotoxicity.

**Supplementary Information:**

The online version contains supplementary material available at 10.1007/s00204-025-04004-4.

## Introduction

Ochratoxin A (OTA), a mycotoxin produced by fungi of the genus *Aspergillu*s and *Penicillium,* is frequently found as a contaminant in a wide variety of foods, including cereals, coffee, beer, spices, fresh and dried fruits, preserved meat and ripened cheese (Schrenk et al. 2020). In Europe, mean chronic exposures ranging from 0.64 to 17.79 ng/kg body weight (bw) per day [minimum lower bound (LB) to maximum upper bound (UB)] have been estimated across dietary surveys and age groups (Schrenk et al. 2020). For highly exposed consumers (95th percentile exposures), chronic dietary exposure estimates range from 2.40 to 51.69 ng/kg bw per day (minimum LB to maximum UB). OTA is a potent nephrotoxin in all animal species tested and has been shown to induce high incidences of renal tumors in rodents at comparatively low doses (NTP 1989; WHO 2002). The mode of action (MoA) underlying OTA carcinogenicity is still not fully understood. In particular, the molecular mechanism underlying OTA genotoxicity (i.e., direct vs. indirect) and the contribution of genetic toxicity to OTA carcinogenicity continue to be a major cause for uncertainty in OTA risk assessment (Schrenk et al. 2020). Results from genotoxicity tests are inconsistent. While OTA was negative in the Ames test, OTA caused gene mutations, single- and double-strand breaks (SSBs and DSBs) and a narrow spectrum of chromosomal damage (chromosome hypercondensation, abnormally separated chromatids, multipolar mitotic spindles, endoreduplications, polyploidy and aneuploidy) in mammalian cells in vitro [summarized in EFSA 2006; Schrenk et al. 2020]. These effects appear to be independent of metabolic activation. In vivo, OTA induces SSBs, DSBs, aberrant mitoses and karyomegaly in the outer stripe of the outer medulla, the target site of OTA renal tumorigenicity. In the absence of evidence for the existence of specific OTA-derived DNA adducts, the European food safety authority (EFSA) considered in its 2006 risk assessment that “DNA damage and genotoxic effects of OTA, measured in various in vivo and in vitro studies are most likely attributable to cellular oxidative damage” and established a tolerable weekly intake (TWI) of 120 ng/kg bw per week (EFSA 2006). However, more recent studies demonstrate weak mutagenic effects in the kidney of rats (Hibi et al. 2011; Kuroda et al. 2014) and of p53^−/−^ but not p53^+/+^ mice (Hibi et al. 2013; Kuroda et al. 2015). The mutational spectrum induced by OTA in rat kidney involved primarily large (1–6 kb) deletions, insertions and base substitutions (Hibi et al. 2011), whereas single base deletions at G/C repetitive sequences, single base substitutions and insertions were detected in p53-deficient mice (Kuroda et al. 2015). In view of the mutational spectrum (including the absence of GC:TA transversions that are typically caused by 8-hydroxy-2′-deoxyguanosine), which is not consistent with reactive oxygen damage, and the lack of evidence for increased formation of 8-hydroxy-2′-deoxyguanosine in rodent kidney in vivo, it appears that OTA-induced mutations are not simply a result of oxidative DNA damage (Hibi et al. 2011; Mally et al. 2005; Schrenk et al. 2020). Consequently, EFSA considered in its recent assessment that the genetic effects of OTA can no longer be attributed to oxidative stress (Schrenk et al. 2020). Taking into account the new data on in vivo genotoxicity of OTA that had become available since the previous assessment in 2006 and the resulting uncertainties regarding the mechanism of OTA genotoxicity, EFSA therefore considered it inappropriate to establish a health-based guidance value and applied a MOE approach (Schrenk et al. 2020). MOEs calculated for neoplastic effects based on a BMDL_10_ of 14.5 µg OTA/kg bw per day for kidney tumors in rats were below 10,000 in most dietary surveys, particularly for breastfed infants and highly exposed consumers, and were thus considered to indicate a health concern (Schrenk et al. 2020). The panel emphasized that the assessment was likely to be overcautious and would rather overestimate the risk associated with dietary intake of OTA but reasoned that the most conservative approach is warranted until the MoA of OTA genotoxicity and carcinogenicity is resolved. Consequently, the panel considered the uncertainties associated with the present assessment as high and recommended further studies elucidating the MoA of OTA genotoxicity/carcinogenicity (Schrenk et al. 2020). In reviewing the available information on the MoA of OTA genotoxicity and carcinogenicity, the EFSA panel concluded that the specific spectrum of chromosomal damage consisting of chromosome hypercondensation, abnormally separated chromatids, multipolar mitotic spindles, endoreduplication, polyploidy and aneuploidy as well as the mutational spectrum induced by OTA in vitro and in vivo were not reconcilable with DNA adducts or oxidative DNA damage. Instead, EFSA considered that these mutations may “arise as a result of the evident replication stress” and that the “abnormal anaphase figures might derive from unresolved ‘replication stress’ with break-induced recombination of DSBs in the early stage of mitosis” (Schrenk et al. 2020).

Replication stress, which is broadly defined as the “slowing or stalling of replication fork progression and/or DNA synthesis”, is increasingly recognized as a major source of genomic instability and cancer (Gaillard et al. 2015; Saxena and Zou 2022; Zeman and Cimprich 2014). The causes of replication stress are numerous, in principle any event that may interfere with the temporally and spatially tightly controlled process of DNA replication, including DNA nicks, gaps, ssDNA, unrepaired DNA lesions (e.g. adducts, cross-links), incorporation of ribonucleotides, overexpression or constitutive activation of oncogenes, interference between replication and transcription, and chromatin inaccessibility (e.g. via reduced histone acetylation) (Zeman and Cimprich 2014). Moreover, decreased supply of essential replication factors is a well-established cause of replication stress. During fork progression, the replisome is in constant need of limiting factors, such as nucleotides (dNTPs) and histones, or their respective chaperones and is thus susceptible to the exhaustion of either factor (Magdalou et al. 2014; Zeman and Cimprich 2014). Stalling of the replisome may result in the accumulation of long stretches of ssDNA due to polymerase-helicase uncoupling, which are highly susceptible to nucleases and other DNA damaging factors (Zeman and Cimprich 2014). Replication stress thus represents a major cause of endogenous DNA damage, which, if unrepaired, may cause both single- and double-strand lesions (Zeman and Cimprich 2014). Moreover, replication stress has been shown to induce a wide spectrum of genetic damage ranging from point mutations to larger chromosomal rearrangements and chromosome segregation defects (Zheng et al. 2016). Chromosome segregation errors are thought to occur as a result of incompletely replicated DNA and formation of anaphase bridges (Gelot et al. 2015). When chromatin bridges are not resolved in anaphase, they are entrapped within the cleavage furrow and likely trigger the abscission checkpoint. From there, cells might abort abscission, resulting in polyploidy and aneuploidy. Alternatively, chromatin bridges might be broken and the consequences of inappropriate repair of these lesions might lead to deletions and translocations (Bizard and Hickson 2018). Moreover, replication stress may cause genetic instability on a genome-wide scale through favoring centrosome amplification and multipolar mitosis (Gelot et al. 2015; Magdalou et al. 2014).

While replication stress may therefore provide a mechanistically plausible explanation for gene mutations, chromosomal damage and mitotic aberrations induced by OTA (Schrenk et al. 2020), experimental evidence for OTA-mediated replication stress is lacking. With the overall objective of addressing uncertainties in the current risk assessment of OTA in food by further elucidating the mechanisms underlying OTA-induced genetic damage, the aim of the present work was to test the hypothesis that OTA interferes with DNA replication, to characterize the cellular response to OTA-mediated replication stress and to experimentally support a mechanistic link between replication stress, mitotic aberrations and genetic damage induced by OTA.

## Material and methods

### Antibodies, chemicals, and reagents

Primary antibodies against p-Histone H2A.X (S139) (20E3), p-Chk1 (S345) (133D3), p-Chk1 (S317) (D12H3 XP), p-ATR (T1989) (D5K8W), p-ATM (S1981) (D6H9), ATR (E1S3S), ATM (D2E2), p-Chk2 (T68) (C13C1), 53BP1 [E7N5D XP (R)], p-Wee1 (S642) (D47G5), p-CDC25C (S216), p-DNA-PKcs (S2056) (E9J4G), DNA-PKcs and GAPDH (D16H11) were obtained from Cell Signaling Technology Europe B.V. (Leiden, the Netherlands). Anti-BrdU B44 mouse IgG and anti-BrdU rat IgG [BU1/75(ICR1) for detection of 5-iodo-2′-deoxyuridine (IdU) and 5-chloro-2′-deoxyuridine (CldU) in the DNA fiber assay were from BD Biosciences (San Jose, USA, Cat. No. 347580) and Abcam (Amsterdam, the Netherlands, Cat. No. ab6326), respectively. Secondary antibodies used for DNA fiber analysis, i.e., (goat anti-mouse IgG (H + L) FITC) and (goat anti-rat IgG (H + L) Alexa Fluor 594), were obtained from Southern Biotech (Birmingham, AL, USA, Cat. No. 1031-02) and Thermo Fisher Scientific Life Technologies GmbH (Darmstadt, Germany, Cat. No. A-11007), respectively. Goat anti-rabbit IgG FITC used for immunofluorescence analysis was purchased from Southern Biotech (Cat. No. 4030-02). The secondary antibody (goat anti-rabbit IgG HRP-linked) employed for western blot analysis was obtained from Cell Signaling Technology (Cat. No. 7074). 5-Iodo-2′-deoxyuridine (IdU) (CAS No. 54-42-2) and 5-chloro-2′-deoxyuridine (CldU) (CAS No. 50–90-8) were purchased from Thermo Fisher Scientific (Cat. No. 122350050) and Sigma-Aldrich (Taufkirchen, Germany, Cat. No. C6891), respectively. 5-Ethynyl-2′-deoxyuridine (EdU) (CAS No. 61135-339, Cat. No. E1057) was obtained from TCI Deutschland GmbH (Eschborn, Germany). Ochratoxin A (OTA) (CAS No. 303-47-9, Cat. No. sc-202749), hydroxyurea (HU) (CAS No. 127-07-1, Cat. No. H8627), cisplatin (CP) (CAS No. 15663-27 1, Cat. No. P4394), aphidicolin (APH) (CAS No. 38966-21-1, Cat. No. A0781) and camptothecin (CPT) (CAS No. 7689-03-4, Cat. No. C9911) were from Sigma-Aldrich. The ATM inhibitor KU-55933 (CAS No. 587871-26-9) was obtained from Cayman Chemical Company (distributed by Biomol GmbH, Hamburg, Germany, Cat. No. 16336). The ATR inhibitor VE-821 (CAS No. 1232410-49-9) was purchased from Sigma-Aldrich (Cat No. SML1415). The DNA-PKcs inhibitor NU-7026 (CAS No. 154447-35-5) was from Abcam (Cat. No. ab120970). Thymidine (CAS No. 50–89-5) was purchased from Sigma Aldrich (Cat. No. T9250). The CDK1 inhibitor RO3306 (CAS No. 872573-93-8) was obtained from Sigma-Aldrich (Cat. No. SML0569). Unless otherwise stated, all other reagents were from Sigma-Aldrich (Taufkirchen, Germany) or Carl Roth GmbH (Karlsruhe, Germany).

### Cell culture and treatment of cells

HK-2 cells (American Type Culture Collection, Manassas, USA) were cultured under standard cell culture conditions (37 °C, 5% CO_2_) in Dulbecco’s modified Eagle’s medium and Ham´s F12 (1:1) (Sigma-Aldrich, Cat. No. D6421) supplemented with 10% fetal bovine serum (Merck Millipore, Darmstadt, Germany, Cat. No. S0615), 2 mM GlutaMAX^™^-I (Thermo Fisher Scientific, Cat. No. 35050038) and 1 × penicillin/streptomycin (Sigma-Aldrich, Cat. No. P4333). Fur subcultivation, 2 × 10^6^ cells were seeded into 75 cm^2^ cell culture flasks. For cell treatment, ochratoxin A (OTA) was freshly dissolved in ethanol. Hydroxyurea (HU) was dissolved in ddH_2_O. Aphidicolin (APH), camptothecin (CPT), 5-ethynyl-2′-deoxyuridine (EdU), the CDK1 inhibitor RO3306 and protein kinase inhibitors (KU-55933, VE-821, NU-7026) were dissolved in DMSO. Cisplatin (CP) was dissolved in 0.9% NaCl. 5-Iodo-2′-deoxyuridine (IdU) and 5-chloro-2′-deoxyuridine (CldU) were dissolved in 1xPBS. Thymidine was dissolved in ddH_2_0. Solutions were added to cell cultures, whereby the final concentration of solvent did not exceed 0.5%. Treatment with solvent served as control.

### DNA fiber assay

The DNA fiber assay was performed according to a published protocol with minor modification (Schwab and Niedzwiedz 2011). HK-2 cells (4.5 × 10^5^ in 3 ml medium per well) were seeded into a six-well plate and left to incubate for 20 h under standard conditions. IdU was added to exponentially growing cells at a final concentration of 25 µM. Cells were incubated for 20 min at 37 °C and 5% CO_2_ before OTA was added to cell cultures for 60 min. After OTA treatment, medium was removed and replaced by fresh medium containing CldU at a final concentration of 250 µM. After 40 min of labeling with CldU, cells were washed once with ice-cold 1xPBS (pH 7.4), trypsinized and resuspended at a concentration of 7.5 × 10^5^ cells/ml in 1xPBS. Labeled cells were kept on ice. 2 µl of the cell suspensions was spotted next to the frosted end on a microscopic glass slide (Elka Frosted Edge, Cat. No. 42406100, Glaswarenfabrik Karl Hecht GmbH & Co. KG) and left to air-dry until the volume of the drop was greatly reduced but not dry (~ 4 min.). 7 µl of lysis solution (50 mM EDTA, 0.5% SDS, 200 mM Tris–HCl pH 7.5) was pipetted on top of the cell suspension and gently mixed by stirring with the pipette tip. The suspension was left to incubate for exactly 2 min for cell lysis to proceed. Slides were manually tilted to an angle of about 30–45 °C to spread DNA fibers by gravity and were then placed horizontally again to air-dry. Glass slides were immersed in a 3:1 methanol/acetic acid fixation solution for 10 min. Slides were briefly washed in distilled water and incubated for 80 min in 2.5 M HCl to denature and depurinate DNA. Samples were then washed 3 times with 1xPBS and incubated for 20 min in blocking buffer (5% BSA in 1xPBS) at room temperature (RT). Primary anti-BrdU antibodies were diluted 1:25 (mouse) and 1:400 (rat) in blocking solution and pipetted onto the slides for 2 h at RT. Slides were washed with 1xPBS before secondary antibodies (1:500 goat anti-mouse and 1:400 goat anti-rat) were pipetted onto the slides for 1 h at RT in the dark. Before mounting medium was applied (Vectashield, Biozol Diagnostica Vertrieb, Eching, Germany, Cat. No. VEC-H-1000), slides were again washed with 1xPBS (3 × 5 min). Sealed slides were stored at − 20 °C until analysis. Images were acquired by confocal laser scanning microscopy (microscope: Leica TCS SP5, lens: DEMI6000 Leica HCX PL APO lambda blue 63.Dx 1.40 OIL UV, Leica Microsystems GmbH, Wetzlar, Germany) and analyzed in ImageJ. For each sample, the length of at least 150 fibers (distributed over 10–15 images across the slide) was determined. Fiber lengths were converted from µm into kb using a conversion factor of 2.59 kb/µm reported for DNA fibers spread by gravity (Jackson and Pombo 1998).

### Extended chromatin fiber assay

The extended chromatin fiber assay was performed as previously described (Dunleavy 2012) with minor modification. HK-2 cells (2.7 × 10^5^ in 3 ml medium per well) were seeded into a six-well plate and incubated for 24 h under standard conditions. OTA was added to exponentially growing cells for 60 min, before cells were washed once with 1xPBS and labeled with EdU (10 µM) for 30 min to mark newly replicating DNA. Cells were again washed with 1xPBS, trypsinized and resuspended at a concentration of 7.5 × 10^5^ cells/ml in 0.5% sodium citrate. Cell suspensions were vortexed, left to incubate for 15 min at RT before being vortexed again. 100 µl of cell suspension was cytospun onto superfrost microscopic slides (OTS, A. Hartenstein Laborbedarf GmbH, Würzburg, Germany) at 800 rpm for 4 min using slotted funnels. After cytospinning, slides were immersed into lysis buffer (10 mM Tris pH 7.5, 1% Triton-X-100, 500 mM NaCl, 500 mM Urea) and left to incubate for 25 min at RT. Slides were slowly and steadily removed from the lysis buffer by hand to achieve stretching of chromatin fibers via surface tension. Slides were immediately fixed in 4% paraformaldehyde (PFA) for 10 min before they were washed twice in 1xPBS and once in 1xPBS + 0.1% Triton-X-100. EdU detection was performed for 30 min at RT using the Click-iT^®^-EdU Imaging Kit Alexa Fluor^™^ 594 dye (Invitrogen by Thermo Fisher Scientific, Eugene, Oregon, USA, Cat. No. 10339). Blocking solution (1xPBS + 5% BSA + 0.1% Triton-X-100) was pipetted onto slides and left to incubate for 30 min at RT. Incubations with primary and secondary antibodies were performed as described for the DNA fiber assay. Slides were mounted with Vectashield mounting medium (Vectashield antifade mounting medium with DAPI, Biozol Diagnostica Vertrieb, Eching, Germany, Cat. No. VEC-H-1200). Sealed slides were stored at − 20 °C until analysis. Images were acquired by confocal laser scanning microscopy (microscope: Leica TCS SP5, lens: DEMI6000 Leica HCX PL APO lambda blue 63.Dx 1.40 OIL UV, Leica Microsystems GmbH, Wetzlar, Germany).

### Western blot analysis

Exponentially growing HK-2 cells were treated with OTA and HU as described above. After 1 and 4 h of treatment, cells were washed with 1xPBS, trypsinized and resuspended in 1xPBS. Cells were centrifuged for 5 min at 10,000 rpm at 4 °C before cell pellets were roughly resuspended in RIPA buffer (50 mM Tris, 150 mM NaCl, 0.1% SDS, 1% Triton-X-100, 0.5% sodium deoxycholate, 1 mM NaF, 1 mM Na_3_VO_4_, freshly added 1 mM EDTA and 1 × Halt^™^ Protease-Inhibitor-Cocktail, Thermo Fisher Scientific, Cat. No. 87785) and left to incubate for 20 min on ice for cell lysis. Samples were centrifuged for 15 min at 10,000 rpm. Supernatants were transferred into fresh tubes and protein concentrations were determined using the Pierce^™^ BCA Protein Assay Kit (Thermo Fisher Scientific). Depending on protein size, proteins (20 µg) were separated on 5 or 10% sodium dodecyl sulfate (SDS)-polyacrylamide gels and transferred onto polyvinylidene difluoride (PVDF) membranes (Immobilon^®^-FL, Merck Millipore Ltd. Tullagreen, Carrigtwohill, Co. Cork, Ireland). Membranes were blocked in 5% BSA in 1xTBS-T buffer (20 mM Tris pH 7.5, 150 mM NaCl, 0.1% Tween 20) for 1 h at RT and subsequently incubated with primary antibodies overnight at 4 °C. Primary antibodies were diluted 1:1000 in blocking solution. Chemiluminescence detection was performed using HRP-conjugated secondary antibodies (1:2000 in 1xTBS-T), the Clarity Western ECL Substrate Kit (Bio-Rad Laboratories Inc., Munich, Germany, Cat. No. 1705061) and the CCD system (ImageQuant^™^ LAS 4000, GE HealthCare, Germany). For protein normalization of p-ATR (T1989), p-ATM (S1981) and p-DNA-PKcs (S2056), western blots of p-ATR (T1989), p-ATM (S1981) and p-DNA-PKcs (S2056) were stripped to reprobe corresponding unmodified kinases. To this end, membranes were washed once with 1xTBS-T. Membranes were then incubated in pre-warmed (~ 50 °C) stripping buffer (2% SDS, 62.5 mM Tris–HCl pH 6.8, 0.8% 2-mercaptoethanol) for 45 min at 50 °C. Membranes were rinsed under running deionized water for 1 h and were then washed five times for 5 min each with 1 × TBS-T. Reprobing of membranes with the antibody against the loading control proteins and chemiluminescence detection were performed as described above. For the remaining proteins, GAPDH (~ 36 kDa) served as loading control. For protein normalization of p-Chk1 (S345, S317) (~ 56 kDa) and p-Chk2 (T68) (~ 62 kDa), the membrane was cut at 40 kDa after blocking using prestained protein molecular weight markers (PeqGOLD, VWR, Cat. No. 27–2110. The upper part of the membrane was incubated in primary antibody solution against p-Chk1 (S345, S317) or p-Chk2 (T68), the lower part in antibody solution against GAPDH. For protein normalization of γH2AX (~ 15 kDa), the membrane was cut at 20 kDa after blocking. The upper part of the membrane was incubated in primary antibody solution against GAPDH and the lower part in primary antibody solution against γH2AX. Western blot bands were quantified using ImageJ (Fiji). Target protein intensities were normalized to loading control protein levels (GAPDH, ATR, ATM, DNA-PKcs) detected on the same membrane and were expressed in relation to respective untreated control samples (0 µM OTA).

### Cell synchronization in late G_2_and OTA treatment during mitosis

HK-2 cells (1.5 × 10^4^ in 200 µl medium per well) were seeded into 8-well chamber slides (ibidi GmbH, Gräfelfing, Germany, Cat. No. 80826) and were left to incubate for 24 h under standard conditions. After reaching 30% confluency, cells were treated with the CDK1 inhibitor RO3306 at a final concentration of 9 µM for 24 h at 37 °C and 5% CO_2_. Cells were washed twice with 1xPBS for 30 s to be released into mitosis. Following release into mitosis, cells were treated with OTA for 4 h before they were fixed and stained for γH2AX as described below.

### Cell synchronization in late G_1_/S and OTA treatment during S phase

Cells were synchronized in late G_1_/S using a double thymidine block. HK-2 cells (1.5 × 10^4^ in 200 µl medium per well) were seeded into 8-well chamber slides and were left to incubate for 24 h under standard conditions. Thymidine was added to exponentially growing cells to a final concentration of 2 mM. After 18 h of incubation at 37 °C and 5% CO_2_, cells were washed once with 1xPBS and were incubated in fresh medium for 9 h to continue cell cycle progression. To arrest all cells in late G_1_/S, a second thymidine block was performed for 18 h. Cells were washed once with 1xPBS and treated with OTA for 4 h during S phase before immunofluorescence analysis was performed. To monitor proper release of cells into S phase, cells were treated with EdU for 4 h at a final concentration of 10 µM. In experiments involving protein kinase inhibitors (KU-55933, VE-821, NU-7026), cells were pretreated with inhibitors during the last hour of the second thymidine block. After release of cells into S phase, inhibitors were added to cells again for 4 h concomitantly with OTA treatment.

### Immunofluorescence analysis

Synchronization and treatment of cells were performed as described above. Cells were washed once with 1xPBS and fixed with 4% PFA in 1xPBS for 15 min at RT. After cells were washed with 3% BSA in 1xPBS for 5 min each, they were permeabilized in 0.5% Triton-X-100 in 1xPBS for 20 min at RT. Cells were washed again with 3% BSA in 1xPBS before EdU detection was performed for 30 min at RT using the Click-iT^®^-EdU Imaging Kit Alexa Fluor^™^ 594 dye (Invitrogen by Thermo Fisher Scientific, Eugene, Oregon, USA, Cat. No. 10339). Cells were washed twice with 3% BSA in 1xPBS for 5 min each before non-specific binding sites were blocked by incubation of cells in 5% BSA, 0.1% Triton-X-100 in 1xPBS for 1 h at RT. Primary antibodies were diluted 1:500 in 1% BSA, 0.1% Triton-X-100 in 1xPBS (p-ATR (T1989), p-ATM (S1981), p-Chk2 (T68), p-CDC25C (S216) and p-Wee1 (S642) were diluted 1:250). Cells were incubated with primary antibody solution overnight at 4 °C in the dark. Cells were washed three times with 1xPBS for 5 min each before the secondary antibody was added to cells for 1 h at RT in the dark. Cells were washed again with 1xPBS (3 × 5 min) and covered with Vectashield mounting medium (Vectashield antifade mounting medium, Biozol Diagnostica Vertrieb, Eching, Germany, Cat. No. VEC-H-1200). Slides were stored at − 20 °C until analysis. Images were acquired by confocal laser scanning microscopy (microscope: Leica TCS SP5, lens: DEMI6000 Leica HCX PL APO lambda blue 63.Dx 1.40 OIL UV, Leica Microsystems GmbH, Wetzlar, Germany). Signal intensities were quantified using ImageJ (Fiji). For each sample, at least ~ 150 cells (distributed over 10–15 images across the well) were analyzed.

## Results

### OTA delays replication fork progression

**Fig. 1:**
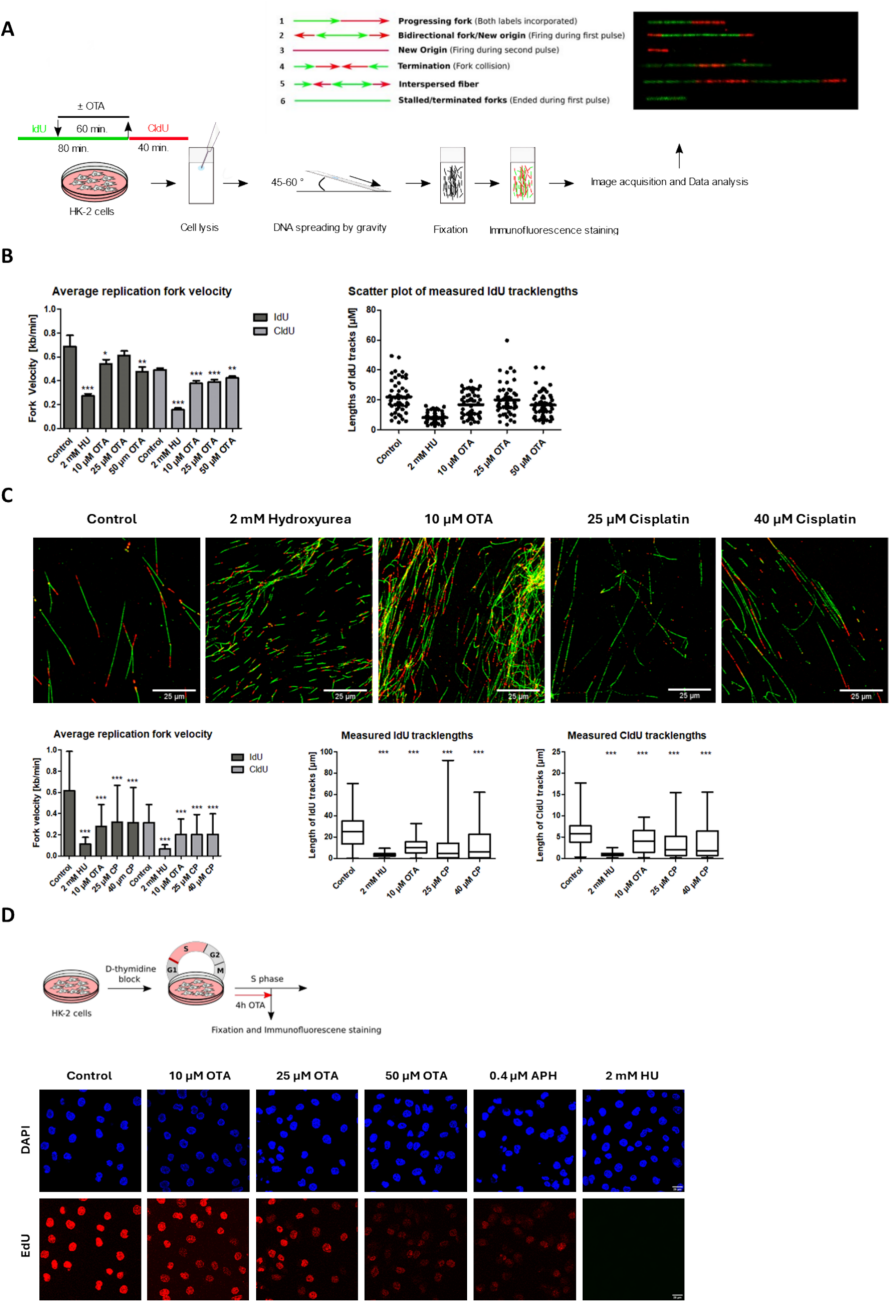
Replication fork dynamics determined in HK-2 cells treated with OTA by DNA fiber analysis and labeling of nascent DNA by EdU.** A** Labeling scheme and different types of replication patterns produced in the DNA fiber assay due to sequential labeling of newly synthesized DNA with IdU (green) and CldU (red). After 20 min of incubation with IdU (25 µM), cells were treated for 1 h with HU (2 mM), OTA (10 µM, 25 µM, 50 µM) or CP (25 µM, 40 µM). Cells were then incubated for 40 min in fresh medium containing CldU (250 µM). Hydroxyurea (HU) which compromises DNA replication through exhaustion of cellular dNTP levels and the DNA crosslinking agent Cisplatin (CP) were included as positive controls. The direction of fork movement can be determined in merged green–red tracks to reveal (1) actively replicating forks, (2) bidirectional replication from origins during the 1st label, (3) replication from origins during the 2nd label (red only), (4) terminations, (5) interspersed fibers and (6) stalled forks (green only). **B** Left: Average replication fork velocity during the first (IdU) and second (CldU) labeling period. Data are presented as means ± SD of three biological replicates (n = 3). In each experiment, ~ 200 IdU and CldU tracks were measured per sample. Statistically significant changes compared to controls were determined by one-way ANOVA with Dunnett´s post hoc test (*p ≤ 0.05, **p ≤ 0.01, ***p ≤ 0.001). Right: Scatter plot of measured IdU tracklengths representative of three biological replicates (n = 3). 52 random tracks out of 200 measured ones are shown per sample. Black line represents the mean value. **C** From top to bottom. Representative immunofluorescence images of labeled DNA fibers generated in HK-2 cells exposed to OTA as compared to HU and CP (Scale bar = 25 µm). In response to HU and OTA a global reduction in tracklengths is evident. In contrast, tracklengths of cells treated with CP span the same range as in controls, with individual tracks drastically shortened, representing replication forks proximal to DNA lesions. Images were uniformly adjusted for brightness and contrast for better visualization. Average replication fork velocity during IdU and CldU labeling periods and box–whisker blots of measured IdU and CldU tracklengths. Note that tracks lacking the second label observed in cell treated with HU and CP are not depicted in the blots. Statistically significant changes compared to controls by one-way ANOVA with Dunnett´s post hoc test are indicated (*p ≤ 0.05, **p ≤ 0.01, ***p ≤ 0.001). In addition, the Mann–Whitney U test, a non-parametric test that compares the distribution of two unmatched groups was used. Of note, significant changes in the distribution of tracklengths were observed between OTA and CP treatment groups (IdU: OTA vs. 25 µM CP, p ≤ 0.001; CldU: OTA vs. 25 µM CP, p ≤ 0.001; OTA vs. 40 µM CP, p ≤ 0.01). **D** Immunofluorescence analysis of EdU in HK-2 cells exposed to OTA for 4 h during S phase, showing a concentration-dependent decrease in EdU incorporation. After HK-2 cells were arrested in G_1_/S by double thymidine block they were treated with OTA (10 µM, 25 µM, 50 µM), APH (0.4 µM) or HU (2 mM) for 4 h during S phase and concomitantly labeled with EdU (10 µM). APH (DNA polymerase inhibitor) and HU (ribonucleotide reductase inhibitor) were employed as positive controls. Images acquired by confocal laser scanning microscopy are representative of three biological replicates (n = 3)

We used the DNA fiber assay to test the hypothesis that OTA interferes with DNA replication. The DNA fiber assay is an invaluable technique to study replication fork dynamics at single molecule resolution (Quinet et al. 2017). It relies on the sequential pulse-labeling of cells with two different thymidine analogs, such as 5-iodo-2′-deoxyuridine (IdU) and 5-chloro-2′-deoxyuridine (CldU), and subsequent visualization of thymidine analogs incorporated into replicating DNA using immunofluorescence. Confocal imaging of DNA fibers allows for measurement of newly synthesized strand lengths, calculation of replication fork velocity and determination of different replication structures based on the labeling pattern of individual DNA tracks (Fig. 1A).

In a first set of experiments, HK-2 cells were first incubated with IdU for 20 min before OTA was added for 60 min. After 80 min of labeling with IdU, the medium was replaced by fresh medium containing CldU as the second thymidine analog. OTA was applied at concentrations (10–50 µM) slightly above plasma concentrations reported to be achieved in rats under conditions of OTA carcinogenicity (7.42 µM) and previously shown to induce chromosome segregation defects and mitotic aberrations in kidney cells in vitro (Czakai et al. 2011; Limbeck et al. 2018; Rached et al. 2007, 2006). Hydroxyurea (HU), which inhibits replication through depletion of cellular deoxynucleotide triphosphate (dNTP) levels, served as a positive control. As expected, HU (2 mM) resulted in a significant decrease in average fork velocity as compared to untreated cells (Fig. 1B). In cells treated with OTA, a statistically significant reduction in fork velocity was observed at all concentrations tested (Fig. 1B) although the effect was much less pronounced as compared to HU. OTA did not result in replication fork stalling, whereas HU also produced a significant increase in stalled replication forks as evidenced by the lack of CldU (red) labeling of the majority of DNA fibers (Fig. 1C). Essentially the same effects of OTA on replication fork velocity were observed when cells were pretreated with OTA for 60–120 min prior to pulse-labeling with both thymidine analogs (data not shown).

A second set of experiments was designed to understand if the decrease in fork velocity in response to OTA is characterized by global perturbation of DNA replication or rather by DNA lesions that are generated randomly throughout the genome and present obstacles for DNA replication. Thus, the distribution of individual IdU- and CldU tracklengths was determined in cells treated with OTA (10 µM), HU (2 mM) as a compound known to cause global replication stress through depletion of dNTPs, and cisplatin (CP, 25 and 40 µM) as a DNA crosslinking agent that may affect only those replication forks proximal to these lesions.

As expected, HU caused a pronounced, global effect on DNA replication as evidenced by the significant and uniform reduction of tracklengths (Fig. 1C). Box plots of individual IdU and CldU tracklengths revealed a rather dense distribution in cells treated with OTA, comparable to HU, indicating that OTA slows fork progression in a global manner (Fig. 1C). In contrast, tracklengths generated from cells treated with cisplatin (CP) spanned the same range as in controls, with individual tracks drastically shortened, representing perturbation of replication forks proximal to DNA lesions (Fig. 1C).

To provide further evidence that OTA acts primarily in S phase via interference with DNA replication, we arrested cells in late G_1_/S by double thymidine block, followed by treatment with the thymidine analog 5-ethynyl-2′-deoxyuridine (EdU) and OTA for 4 h during S phase (Fig. 1D). In support of the significant effects of OTA on replication fork velocity, immunofluorescence analysis revealed a marked, concentration-dependent decrease in the incorporation of EdU into DNA in the presence of OTA (Fig. 1D).

### γH2AX induction by OTA appears to be linked to DNA replication

**Fig. 2:**
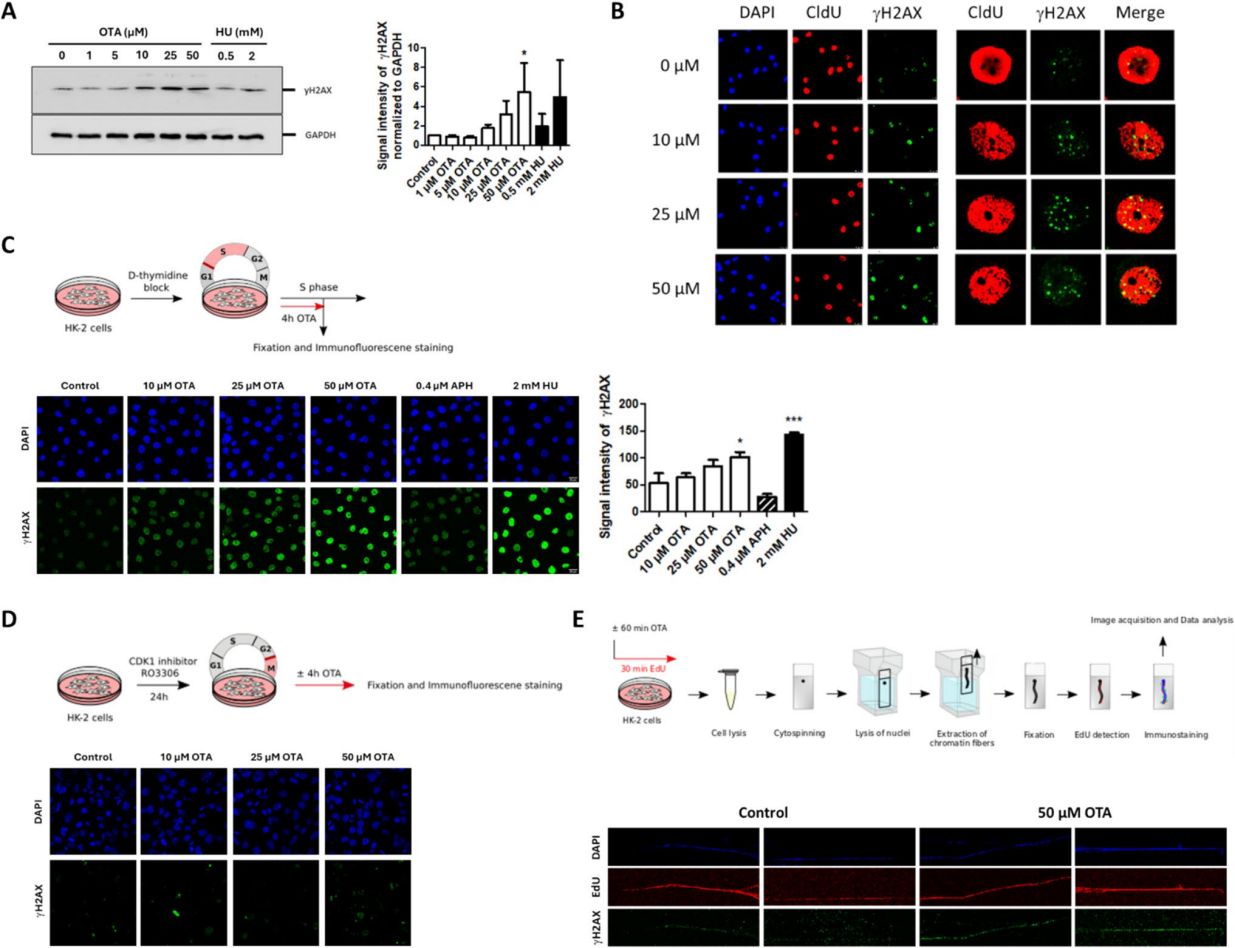
γH2AX induced by OTA appears to be coupled to DNA replication. **A** Western blot analysis of γH2AX in HK-2 cells treated with OTA (1 µM, 5 µM, 10 µM, 25 µM, 50 µM) and HU (0.5 mM, 2 mM) for 4 h, demonstrating a concentration-dependent increase in γH2AX. Shown is one representative result of three independent experiments (n = 3). GAPDH staining served as loading control. Quantitative analysis of western blot results by densitometry confirmed OTA-mediated dose-related γH2AX formation. Data are expressed as means ± SD relative to controls obtained from three biological replicates (n = 3). Statistically significant changes compared to controls were determined by one-way ANOVA with Dunnett´s post hoc test (*p ≤ 0.05). **B** Immunofluorescence analysis of γH2AX in HK-2 treated with OTA (10 µM, 25 µM, 50 µM) for 6 h, confirming a concentration-dependent increase in γH2AX foci. Co-staining of CldU (250 µM) incorporated into cells during S phase suggests replication-coupled γH2AX foci formation. **C** Immunofluorescence analysis of γH2AX in HK-2 cells synchronized in late G_1_/S by double thymidine block and treated with OTA (10 µM, 25 µM, 50 µM), APH (0.4 µM) and HU (2 mM) for 4 h during S phase, highlighting a concentration-related increase in γH2AX by OTA. Shown are representative immunofluorescence images and quantitative analysis of γH2AX. Data are represented as means ± SD of three biological replicates (n = 3). Statistically significant changes compared to controls were determined by one-way ANOVA with Dunnett´s post hoc test (*p ≤ 0.05, **p ≤ 0.01, ***p ≤ 0.001). **D** Immunofluorescence analysis of γH2AX in HK-2 cells arrested in late G_2_ using the CDK1 inhibitor RO3306 and exposed to OTA (10 µM, 25 µM, 50 µM) for 4 h during mitosis, revealing no evidence for an apparent induction of γH2AX by OTA. Results are representative of three independent experiments (n = 3). **E** Visualization of γH2AX along replicative chromatin fibers determined in HK-2 cells treated with OTA for 1 h by extended chromatin fiber analyses, showing an increase in γH2AX along fibers that undergo active DNA replication. Cells were treated with OTA (50 µM) for 1 h before being labeled with EdU (10 µM) for 30 min. Immunofluorescence images show representative fibers obtained in three independent experiments (n = 3). Images shown in **B**, **C**, **D** and **E** were uniformly adjusted for brightness and contrast for better visualization

Phosphorylation of H2AX at serine 139 (γH2AX) is widely used as a marker of DNA double-strand breaks (DSBs), but increasing evidence shows that it is also formed in response to replication stress in the absence of DSBs (Khurana and Oberdoerffer 2015). As OTA has previously been reported to increase γH2AX at the target site of OTA carcinogenicity in vivo (Kuroda et al. 2014; Taniai et al. 2014), we were interested to understand if induction of γH2AX is linked to replication stress mediated by OTA. Using western blot analysis, we first established that 4 h treatment of HK-2 cells with OTA causes a concentration-related increase in γH2AX (Fig. 2A), whereas no clear concentration-related changes were evident after 1 h treatment (Supplemental Fig. S1). Immunofluorescence analysis in cells exposed to OTA revealed γH2AX foci almost exclusively in cells with newly replicated DNA identified via CldU incorporation during S phase, suggesting a link between DNA replication and induction of γH2AX foci (Fig. 2B). In synchronized cells treated with OTA specifically during S phase for 4 h, a clear, concentration-related increase in γH2AX labeling was observed in all cells (Fig. 2C). This effect reached statistical significance at 50 µM OTA. In contrast, no significant effect on γH2AX was observed in cells synchronized in late G_2_ by treatment with the CDK1 inhibitor RO3306 and exposed to OTA for 4 h during mitosis (Fig. 2D). To further support a replication-coupled mechanism of OTA-mediated induction of γH2AX, we employed the extended chromatin fiber assay to visualize γH2AX along individual chromatin fibers at or distant from sites of active DNA replication (Cohen et al. 2009). To mark newly replicating DNA, cells were labeled with EdU. Using this technique, which allows analysis of individual replicative DNA fibers as opposed to western blot analysis of the entire cell population (Supplemental Fig. S1), we observed a clear increase in γH2AX along newly replicating chromatin fibers after only 1 h of exposure to OTA (Fig. 2E). These results provide further evidence for a link between perturbation of DNA replication and γH2AX induction by OTA.

### DNA damage induced by OTA during S phase persists through mitosis

**Fig. 3:**
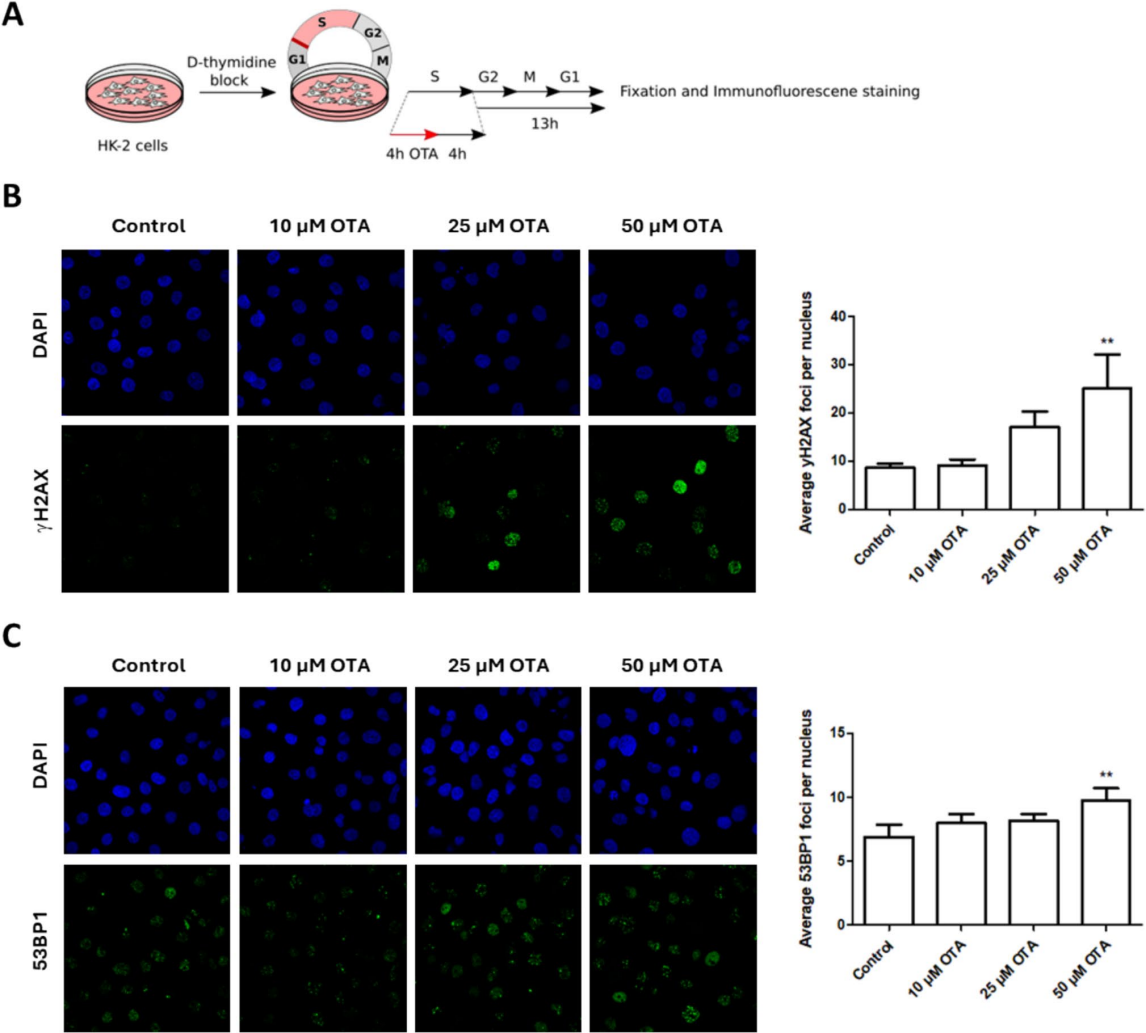
DNA damage induced by OTA during S phase persists through mitosis. **A** Treatment scheme used for immunofluorescence analysis of γH2AX and 53BP1 in G_1_ cells (HK-2) treated with OTA (10 µM; 25 µM, 50 µM) for 4 h during S phase. **B**, **C** Results reveal a concentration-dependent increase in γH2AX and 53BP1 in G_1_ cells exposed to OTA during S phase, indicating that DNA damage generated by OTA during S phase or mitosis might be not fully repaired during mitosis and persists into G_1_ phase. Representative immunofluorescence images and corresponding quantification are representative of three biological replicates (n = 3). Immunofluorescence images were uniformly adjusted for brightness and contrast for better visualization. Data are expressed as means ± SD of three biological replicates (n = 3). Statistically significant changes compared to controls were determined by one-way ANOVA with Dunnett´s post hoc test (*p ≤ 0.05, **p ≤ 0.01)

γH2AX induction was also observed in cells arrested in late G_1_/S by double thymidine block, exposed to OTA during S phase and allowed to continue into mitosis (Fig. 3A and B). Likewise, a small but statistically significant, concentration-dependent increase in 53BP1 was observed in G_1_ cells arrested in late G_1_/S and treated with OTA during S phase (Fig. 3C). 53BP1 nuclear bodies shield DNA damage generated during S phase or mitosis in nuclear compartments to allow for their repair during the next cell cycle. As they are exclusive for G_1_, they serve as ideal biomarkers for DNA DSBs that either arise during or persist through mitosis. The increase in 53BP1 observed in G_1_ cells exposed to OTA during S phase may thus indicate that the DNA damage generated by OTA during S phase is not fully repaired during G_2_ and mitosis and is transmitted to the subsequent G_1_ phase.

### Cellular response to replication stress

Upon replication stress, activation of DNA damage response (DDR) pathways serves to delay cell cycle progression and repair DNA damage before cells enter mitosis in order to limit transmission of DNA damage to daughter cells. However, it has been reported that moderate levels of replication stress (induced by low doses of aphidicolin) may escape checkpoint control (Koundrioukoff et al. 2013). As a result, cells with under-replicated DNA or unresolved DNA damage may continue into mitosis, leading to mitotic defects, such as anaphase bridges, centrosome amplification, and multipolar mitosis, which result in chromosome segregation errors (Gelot et al. 2015). Thus, we were interested to characterize the cellular response to OTA-mediated replication stress by analyzing the stepwise activation of the ATR-Chk1 and ATM-Chk2 DDR pathways.

**Fig. 4:**
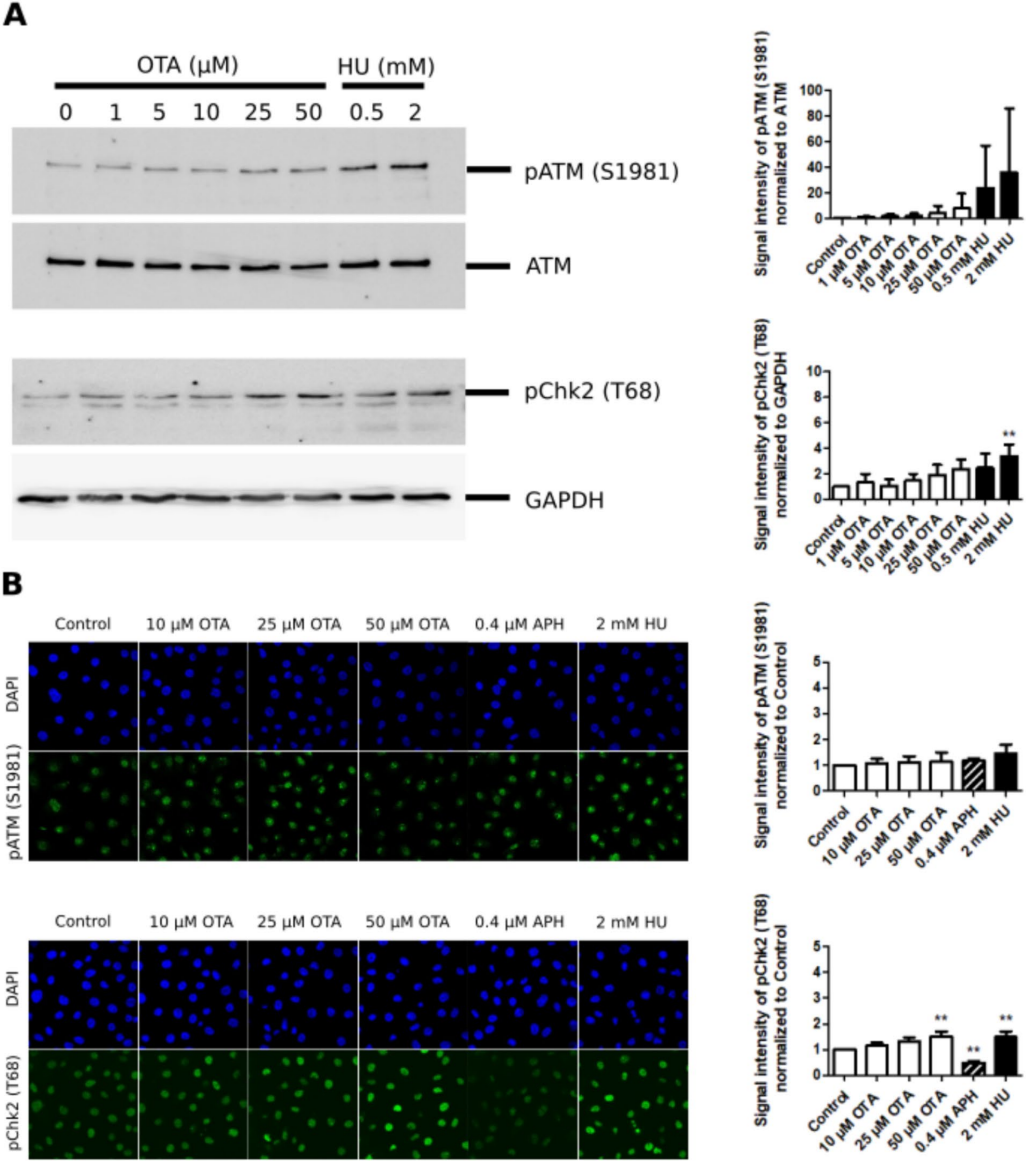
Analysis of ATM-Chk2 DNA damage signaling in response to OTA. **A** Western blot analysis of p-ATM (S1981) and p-Chk2 (T68) in HK-2 cells exposed to OTA (1 µM, 5 µM, 10 µM, 25 µM, 50 µM) and HU (0.5 mM, 2 mM) for 4 h, showing a slight concentration-related but not statistically significant increase in phosphorylation of ATM (S1981) and Chk2 (T68) in response to OTA. Western blot and corresponding quantification of western blot results by densitometry are representative of three independent experiments (n = 3). ATM and GAPDH served as loading controls. Data are expressed as means ± SD relative to controls obtained from three biological replicates (n = 3). Statistically significant changes compared to controls were determined by one-way ANOVA with Dunnett´s post hoc test (*p ≤ 0.05, **p ≤ 0.01). **B** Immunofluorescence analysis of p-ATM (S1981) and p-Chk2 (T68) in HK-2 arrested in late G_1_/S and treated with OTA (10 µM, 25 µM, 50 µM), APH (0.4 µM) and HU (2 mM) for 4 h during S phase. Immunofluorescence images and corresponding quantifications are representative of three biological experiments (n = 3). Images were uniformly adjusted for brightness and contrast for better visualization. Data are represented as means ± SD relative to controls. Statistically significant changes compared to controls were determined by one-way ANOVA with Dunnett´s post hoc test (*p ≤ 0.05, **p ≤ 0.01)

**Fig. 5:**
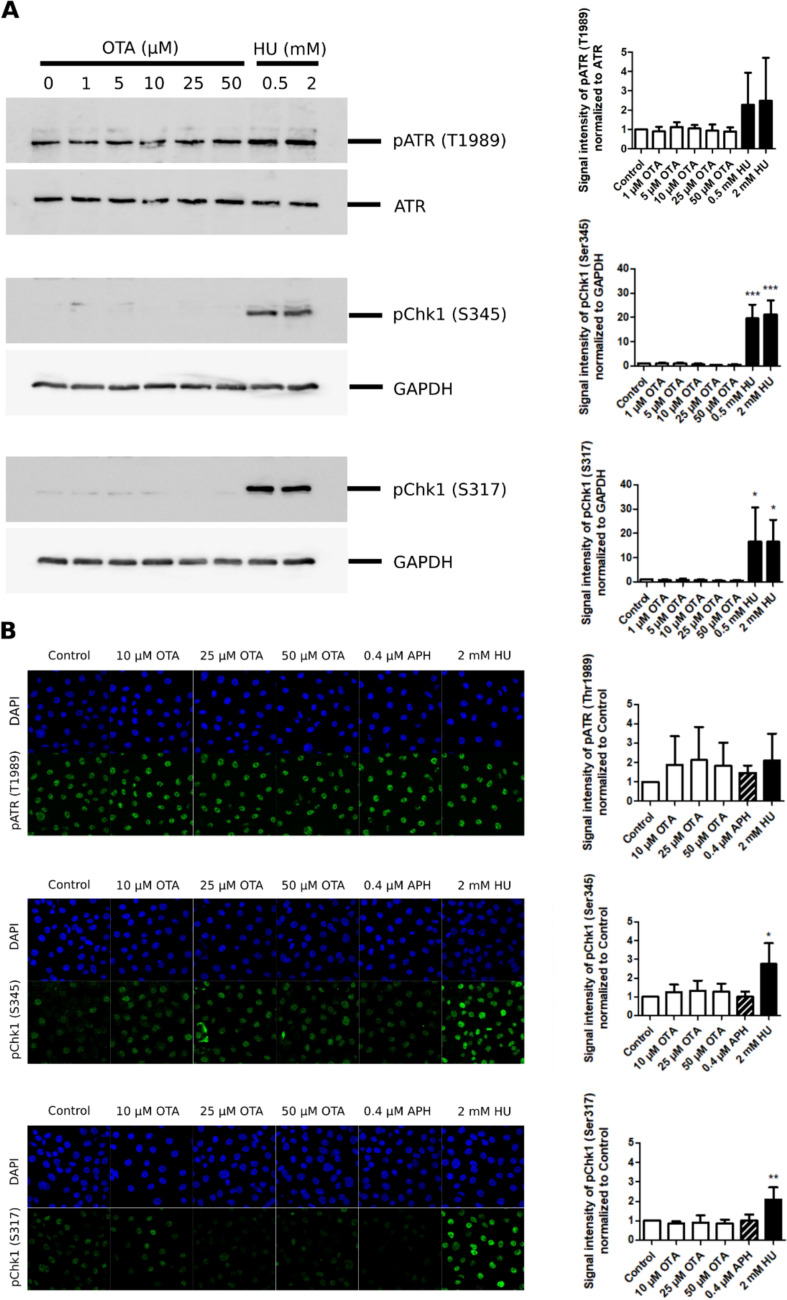
Analysis of ATR-Chk1 signaling in response to OTA. **A** Western blot analysis of p-ATR (T1989), p-Chk1 (S345) and p-Chk1 (S317) in HK-2 cells exposed to OTA (1 µM, 5 µM, 10 µM, 25 µM, 50 µM) and HU (0.5 mM, 2 mM) for 4 h. In contrast to HU, no significant activation of Chk1 is evident in OTA-treated cells. Western blot and corresponding quantification of western blot results by densitometry are representative of three independent experiments (n = 3). ATR and GAPDH served as loading controls. Data are expressed as means ± SD relative to controls obtained from three biological replicates (n = 3). Statistically significant changes compared to controls were determined by one-way ANOVA with Dunnett´s post hoc test (*p ≤ 0.05, **p ≤ 0.01, ***p ≤ 0.001). **B** Immunofluorescence analysis of p-ATR (T1989), p-Chk1 (S345) and p-Chk1 (S317) in HK-2 cells arrested in late G_1_/S and treated with OTA (10 µM, 25 µM, 50 µM), APH (0.4 µM) and HU (2 mM) for 4 h during S phase. Immunofluorescence images and corresponding quantification are representative of three biological experiments (n = 3). Data are represented as means ± SD relative to controls. Statistically significant changes compared to controls were determined by one-way ANOVA with Dunnett´s post hoc test (*p ≤ 0.05, **p ≤ 0.01). Images were uniformly adjusted for brightness and contrast for better visualization

Activation of the ATR-Chk1 and ATM-Chk2 signaling pathways was investigated both by western blot analysis of unsynchronized cells treated for 1–4 h and by immunofluorescence of cells synchronized in G_1_/S and treated with OTA during S phase. Treatment with the positive control HU resulted in a concentration-related increase in phosphorylation of ATM at serine 1981 [p-ATM (S1981)] and Chk2 at threonine 68 [p-Chk2 (T68)] (Fig. 4), suggesting slight activation of the ATM-Chk2 signaling pathway. As expected, more pronounced effects of HU were observed on the ATR-Chk1 DDR pathway, which is most strongly activated when DNA replication is impeded, and ssDNA stretches are formed. This was shown by increased phosphorylation of ATR at threonine 1989 [p-ATR (T1989)] and of Chk1 at serine 345 [p-Chk1 (S345)] and at serine 317 [p-Chk1 (S317)] (Fig. 5).

In contrast to HU, there was no evidence for activation of the ATR-Chk1 DDR pathway by OTA after 1 h and 4 h of treatment (Fig. 5 and Supplemental Fig. S2). Minor effects of OTA were observed on the ATM-Chk2 DDR pathway (Fig. 4 and Supplemental Fig. S2) which is mainly induced in response to DNA double-strand breaks (DSBs) formed as a consequence of DNA damage (Smith et al. 2010). Effectors of the DDR, Wee1 and CDC25C, which are both directly regulated by Chk1 via phosphorylation and control mitotic entry, remained unaffected in cells exposed to OTA during S phase (Supplemental Fig. S3). This may indicate that the DNA replication checkpoint is not fully activated in response to OTA, allowing cells with under-replicated DNA or unresolved DNA damage to continue into mitosis.

### Role of DNA-PKcs in γH2AX induction by OTA

As neither ATR nor ATM appeared to be significantly activated in response to OTA, we were further interested to understand which kinase is involved in γH2AX induction by OTA. We therefore employed selective ATR, ATM and DNA-PKcs inhibitors and treated cells with OTA and the respective inhibitors for 4 h during S phase.

**Fig. 6:**
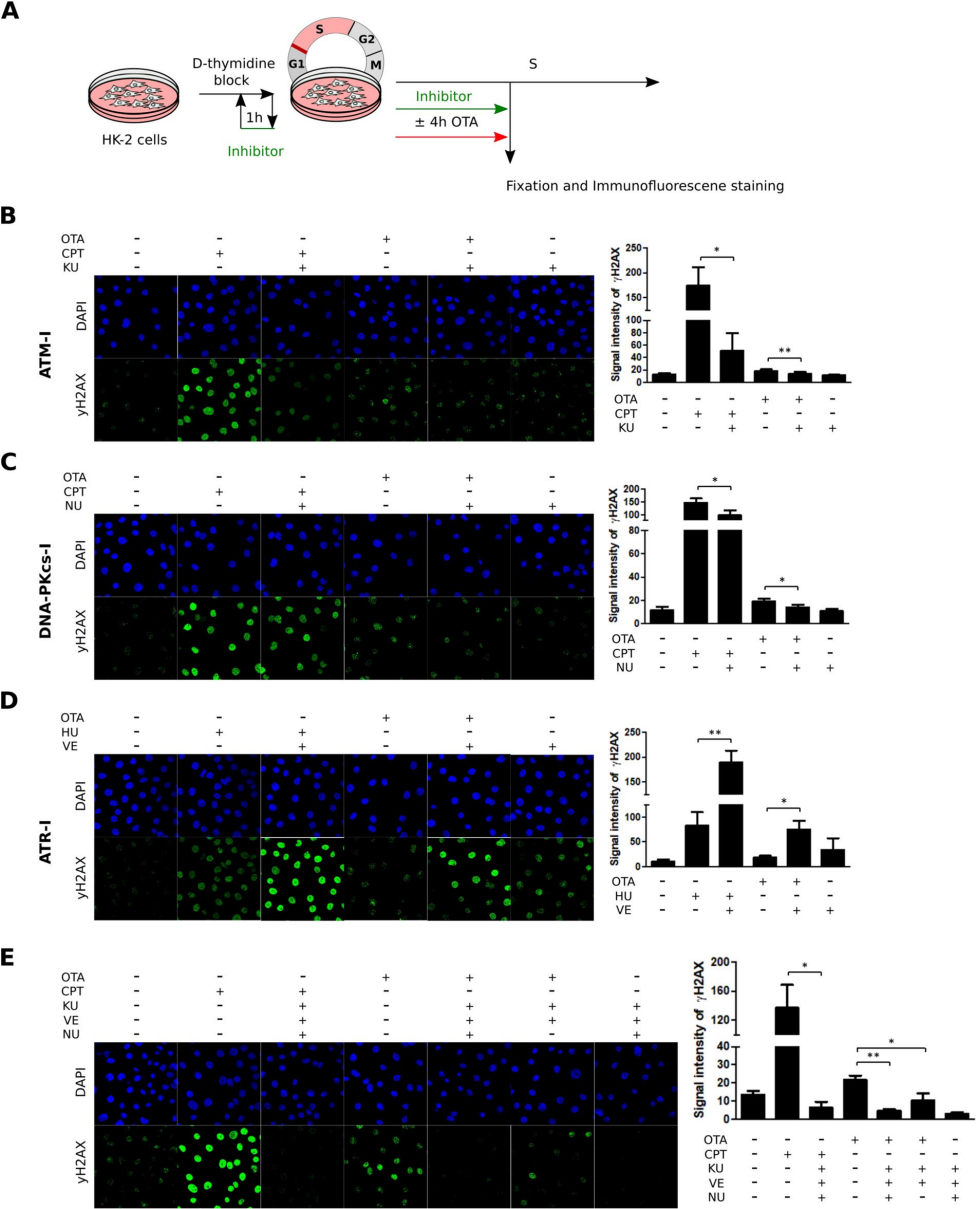
DNA-PKcs seems to be involved in γH2AX induction by OTA. **A** Treatment scheme. HK-2 cells arrested in late G_1_/S were pretreated with inhibitors (40 µM KU-55933, 50 µm NU-7026, 10 µM VE-821) for 1 h during the last hour of the second thymidine block. After release into S phase cells were treated with inhibitors for 4 h alone or concomitantly with OTA (50 µM), CPT (1 µM) or HU (2 mM). **B-E** Immunofluorescence analysis of γH2AX in HK-2 cells treated with OTA ± protein kinase inhibitors. Shown are immunofluorescence images and corresponding quantifications representative of three biological experiments (n = 3). Data are represented as means ± SD. Statistical significance was determined using a paired t test. For clarity of presentation, only statistically significant changes between each treatment group in the absence and presence of the respective kinase inhibitor are shown (*p ≤ 0.05, **p ≤ 0.01). Images were equally adjusted for brightness and contrast for better visualization with exception of CPT- and HU-treated samples (positive controls) in experiments shown in **B**-**E**

The DNA DSB inducing agent, camptothecin (CPT), known to halt relaxation of torsional stress through inhibition of topoisomerase I (Vesela et al. 2017), was chosen as a positive control to test for selective inhibition of ATM (Kim et al. 2011) and DNA-PKcs (Garcia et al. 2014). Analysis by immunofluorescence revealed an apparent increase in γH2AX in cells exposed to CPT, which was significantly reduced in the presence of the ATM (KU-55933) and DNA-PKcs (NU-7026) inhibitors (Fig. 6, B and C). Hydroxyurea (HU), which results in the accumulation of long ssDNA stretches due to polymerase-helicase uncoupling, was selected as a positive control for ATR signaling (Reaper et al. 2011; Vesela et al. 2017). Surprisingly, γH2AX was increased in cells treated with HU and the ATR inhibitor (VE-821) for 4 h during S phase compared to HU treatment alone (Fig. 6D). Similarly, γH2AX was further increased in OTA-treated cells in the presence of the ATR inhibitor VE-821. This suggests that ATR is not primarily responsible for induction of γH2AX induction by OTA in HK-2 cells (Fig. 6D).

In contrast, a slight decrease in γH2AX was observed in cells treated with OTA and the ATM inhibitor KU-55933 compared to OTA treatment alone (Fig. 6B). This is consistent with the minor increase in p-ATM (S1981) and p-Chk2 (T68) in response to OTA and suggests that γH2AX induction may to some extent be mediated by ATM.

**Fig. 7:**
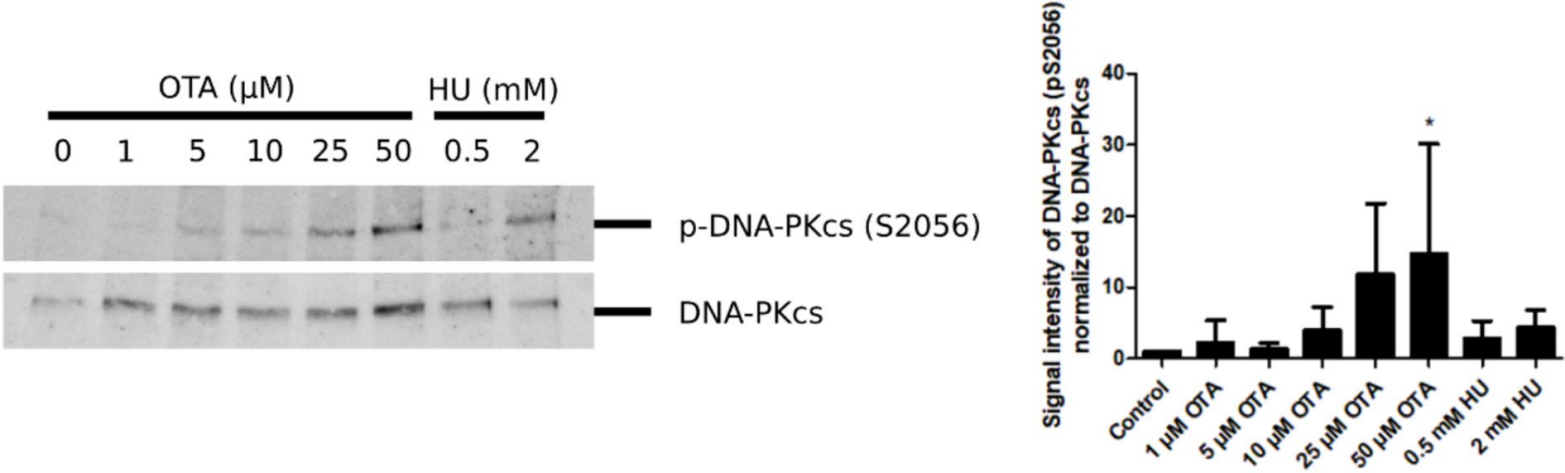
Western blot analysis of p-DNA-PKcs (Ser2056) in HK-2 cells exposed to OTA (1 µM, 5 µM, 10 µM, 25 µM, 50 µM) or HU (0.5 mM, 2 mM) for 4 h. Shown is one representative result of four independent experiments (n = 4) and corresponding quantification. DNA-PKcs served as loading control. Data are expressed as means ± SD relative to controls. Statistically significant changes compared to controls were determined by one-way ANOVA with Dunnett´s post hoc test (* p ≤ 0.05)

Because OTA had no or only minor effects on ATR/ATM signaling, we hypothesized that DNA-PKcs may play a role in phosphorylation of H2AX. Immunofluorescence analysis of cells treated with OTA in the presence of the DNA-PKcs inhibitor NU-7026 revealed a clear reduction of γH2AX signal intensity compared to OTA (Fig. 6C). The apparent involvement of DNA-PKcs in γH2AX induction by OTA was further supported by complete absence of γH2AX in cells treated with OTA and all three kinase inhibitors (i.e., KU-55933, VE-821 and NU-7026) compared to co-treatment with ATM/ATR kinase inhibitors (i.e., KU-55933 and VE-821) only (Fig. 6E) and a concentration-related increase in DNA-PKcs phosphorylation [p-DNA-PKcs (Ser2056)] (Fig. 7).

## Discussion

The overall objective of the present study was to further understand the mechanism of OTA carcinogenicity and genotoxicity to support risk assessment of this important food contaminant. Based on our previous work, we consider that disruption of mitosis coupled with compensatory stimulation of cell proliferation present key events that drive genetic instability and tumorigenesis in kidneys of rats exposed to OTA (Mally 2012; Rached et al. 2007, 2006). However, the molecular events preceding perturbation of mitosis by OTA and the mechanism underlying OTA genotoxicity still need to be resolved. Since both mitotic aberrations and the specific spectrum of mutations and genetic damage induced by OTA can be reconciled with replication stress (Schrenk et al. 2020), we aimed to investigate if perturbation of the S phase replisome machinery by OTA plays a role in OTA genotoxicity.

The significant, OTA-mediated delay in replication fork progression and marked concentration-related decrease in the incorporation of the thymidine analog EdU into DNA of cells treated with OTA during S phase provide clear evidence that OTA at concentrations relevant to OTA carcinogenicity in rat kidney interferes with DNA replication. Importantly, effects of OTA on replication fork velocity were observed after only 1 h OTA treatment, indicating that perturbation of DNA replication by OTA is indeed an early event. Although we do not yet understand the mechanism of replication stress induced by OTA, the observation that all replication forks were affected upon treatment with OTA suggests that OTA acts in a global manner rather than via DNA adducts that present physical obstacles which perturb only replisomes proximal to these lesions. This is consistent with the lack of conclusive evidence for a DNA-reactive MoA of OTA carcinogenicity (EFSA 2006; Mally 2012; Schrenk et al. 2020). Based on previous data demonstrating histone hypoacetylation and overcondensation of mitotic chromosomes in renal epithelial cells treated with OTA (Czakai et al. 2011), we speculate that chromatin inaccessibility or decreased histone supply may play a role.

H2AX phosphorylation at serine 139 (γH2AX) is widely used as a sensitive marker of DNA double-strand breaks but is also increasingly recognized as a marker of replication stress independent of DNA damage (Fragkos et al. 2023). Since OTA has previously been shown to increase γH2AX in vitro and in rodent kidney in vivo (Cui et al. 2013; Kuroda et al. 2014, 2015; Lian et al. 2014; Liu et al. 2012; Taniai et al. 2012; Yang et al. 2014), leading to the conclusion that OTA induces DNA double-strand breaks (Kuroda et al. 2014, 2015), we were interested to determine if γH2AX formation by OTA is linked to replication stress induced by OTA. Results obtained through a combination of complementary techniques demonstrate not only a rapid, concentration-related increase in γH2AX in kidney cells exposed to OTA but also indicate that phosphorylation of H2AX occurs in a replication-coupled manner. Immunofluorescence revealed the presence of γH2AX predominantly in cells that had undergone DNA synthesis as evidenced by co-staining of γH2AX and CldU incorporated into cells during S phase. In synchronized cells, a clear-concentration-related increase in γH2AX was observed when cells were treated with OTA during S phase, but not during M phase. Finally, the extended DNA fiber assay, which allows visualization of proteins in relation to sites of DNA replication along extended chromatin fibers, revealed γH2AX at newly replicated DNA fibers.

Replication stress and DNA damage generated during S phase typically lead to cell cycle arrest through ATR and ATM-mediated checkpoint activation to mediate DNA repair before cells enter mitosis. In the present study, however, both western blot and immunocytochemical analysis of synchronized cells treated with OTA during S phase indicate that the DNA replication checkpoint depending on ATR-Chk1 signaling is not activated in response to OTA. Similarly, only mild activation of ATM-Chk2 signaling was observed. The failure to efficiently activate cell cycle checkpoints despite the clear impact on DNA replication may allow cells with damaged or under-replicated DNA to reach mitosis, jeopardizing chromosome segregation, and in turn genomic integrity (Gelot et al. 2015; Koundrioukoff et al. 2013). In support of this, Koundrioukoff et al. demonstrated that moderate levels of replication stress in response to low concentrations of aphidicolin lead to chromatin loading of sensors and mediators of the ATR pathway but failed to activate Chk1, p53, ATM, Chk2 and RPA2 (Koundrioukoff et al. 2013). Thus, a certain threshold of damage appears to be needed to effectively activate the DDR response and checkpoint-mediated cell cycle arrest (Gelot et al. 2015; Koundrioukoff et al. 2013).

Thus, it appears that HK-2 cells experiencing moderate replication stress in response to OTA escape checkpoint control and proceed into mitosis. During mitosis, under-replicated, intertwined or damaged DNA regions derived from unresolved replication stress may lead to the formation of anaphase bridges, jeopardizing disjunction of sister chromatids. While the formation of ultrafine anaphase bridges (UFBs) by OTA has not yet been experimentally demonstrated, OTA has been shown to induce abnormal anaphase figures sometimes still connected by chromatin bridges (Rached et al. 2006). Persistent anaphase bridges can break, leading to gross chromosomal rearrangements and, in turn, to increasing genomic instability (Umbreit et al. 2020). Furthermore, DNA damage that failed mitotic rescue and bypasses mitosis results in the formation of 53BP1 bodies and/or micronuclei (MN) during G_1_ (Gelot et al. 2015). 53BP1 bodies marked by the p53-binding protein 1 (53BP1) are thought to shield non-repaired DNA damage against secondary damage in nuclear compartments to allow for their repair during the next cell cycle. Results from this study demonstrate a significant concentration-related increase in 53BP1 in cells treated with OTA during S phase and allowed to continue into mitosis. This indicates that DNA damage generated by OTA during S phase is transmitted to mitosis and is insufficiently repaired during mitosis, leading to 53BP1 body formation in the subsequent G_1_ or pseudo-G_1_ phase. Replicative or repair DNA synthesis during early mitosis (MiDAS) has been postulated as a last attempt to complete genome duplication and prevent chromosome segregation defects and mitotic catastrophe (Garribba et al. 2018; Minocherhomji et al. 2015). Although some reports suggest MiDAS can be detected in prometaphase cells by synchronization of cells in late G_2_ followed by mitotic release and subsequent incubation with EdU, we were unable to detect EdU foci that would be indicative of MiDAS in HK-2 cells as well as several other cell lines (RPTEC/TERT1, IHKE, HeLa) in response to OTA and aphidicolin as positive control (Garribba et al. 2018; Minocherhomji et al. 2015). It is possible that the failure to experimentally demonstrate MiDAS may be due to technical challenges. However, there has recently been a controversial discussion whether MiDAS is strictly mitosis-specific based on evidence that cells continuously rescue under-replicated regions from S phase throughout G_2_ to early mitosis (Mocanu et al. 2022).

Phosphorylation of H2AX (γH2AX) can be induced by three major DNA damage kinases, i.e., ATR, ATM and DNA-PKcs (Blackford and Jackson 2017; Podhorecka et al. 2010). Since OTA did not appear to cause significant activation of ATR and ATM, we were interested to understand which kinase in primarily responsible for the induction of γH2AX. Experiments using selective pharmacological inhibitors of ATR, ATM and DNA-PKcs suggested a prominent role of DNA-PKs in phosphorylation of H2AX by OTA, which was further supported by a concentration-related increase in DNA-PK phosphorylation (Ser2056). While the role of DNA-PKcs in activating non-homologous end joining (NHEJ) repair following replication-independent DNA lesions is well defined, its function in response to DNA DSBs during replication has not yet been fully elucidated (Collis et al. 2005; Shimura et al. 2007; Yue et al. 2020). Shimura et al. reported that DNA breaks induced upon mild replication stress were rapidly repaired in cells containing a functional DNA-PKcs, but persisted in DNA-PKcs-deficient cells (Shimura et al. 2007). While cells containing a functional DNA-PKcs did not arrest cell cycle progression and continued DNA synthesis in a delayed manner in the presence of low levels of DNA polymerase inhibition, cells deficient in DNA-PKcs activity halted cell cycle progression through ATR-Chk1-mediated activation of the S phase checkpoint (Shimura et al. 2007). The authors of the study suggested that DNA-PKcs contributes to immediate repair of DNA breaks through delaying DNA replication and increases the threshold for ATR-Chk1-mediated S phase checkpoint activation via preventing Chk1 phosphorylation (Shimura et al. 2007). This crosstalk between DNA-PKcs and ATR-Chk1 signaling may explain the absence of DDR activation in the presence of OTA, which may allow cells with incompletely replicated DNA to enter into mitosis. At the same time, DNA damage repair by DNA-PKcs does not appear to be sufficient to completely resolve the damage induced by OTA during S phase.

Results from this study provide first experimental proof for interference with DNA replication as an early key event in OTA genotoxicity, supporting the hypothesis that mitotic aberrations and genetic damage induced by OTA may arise from unresolved replication stress. Understanding the molecular cause of replication stress by OTA and confirmation of key molecular changes identified in vitro at the target site of OTA carcinogenicity in rat kidney (i.e., OSOM), including characterization of their dose–response may help refine human risk assessment.

## Supplementary Information

Below is the link to the electronic supplementary material.

Supplementary file.1

## Data Availability

All data supporting the findings of this study are available within the paper and its Supplementary Information. Raw data are available from the corresponding author upon reasonable request

## Abbreviations

53BP1: P53-binding protein 1
8-OHdG: 8-Hydroxy-2′-deoxyguanosine
APH: Aphidicolin
ATM: Ataxia–telangiectasia mutated protein kinase
ATR: Ataxia–Telangiectasia and Rad3-related protein kinase
BMDL_10_: Benchmark dose lower confidence limit 10%
BSA: Bovine serum albumin
Bw: Body weight
CDK1: Cyclin-dependent kinase 1
Chk1: Checkpoint kinase 1
Chk2: Checkpoint kinase 2
CldU: 5-Chloro-2′-deoxyuridine
CP: Cisplatin
CPT: Camptothecin
DAPI: 4′,6-Diamidino-2-phenyl-indol-dihydrochloride
DDR: DNA damage response
DMSO: Dimethyl sulfoxide
DNA-PKcs: DNA-dependent protein kinase catalytic subunit
dNTP: Deoxyribonucleotide triphosphate
DSB: DNA double-strand break
EdU: 5-Ethynyl-2′-deoxyuridine
EFSA: European food safety authority
HK-2: Human kidney cells
HU: Hydroxyurea
IdU: 5-Iodo-2′-deoxyuridine
IHKE: Immortalized human kidney epithelial-1 cells
MiDAS: Mitotic DNA synthesis
MoA: Mode of action
MOE: Margin of exposure
OTA: Ochratoxin A
PBS: Phosphate-buffered saline
PFA: Paraformaldehyde
RPA: Replication protein A
RPTEC/TERT1: Renal proximal tubular epithelial cells (immortalized by stable ectopic expression of the human telomerase reverse transcriptase (hTERT)
RT: Room temperature
SSB: DNA single-strand break
ssDNA: Single-stranded DNA
TWI: Tolerable weekly intake
γH2AX: Ser139 phosphorylated H2AX
