# Supplementary material for: Replication stress: an early key event in ochratoxin a genotoxicity?
Source: Arch Toxicol. 2025 Mar 10;99(6):2577–94. doi: 10.1007/s00204-025-04004-4 (PMC12185617; doi:10.1007/s00204-025-04004-4)
Supplement: Supplementary file 1 — Supplementary file.1 [file 204_2025_4004_MOESM1_ESM.docx]

**Supplemental Figure 1**

**
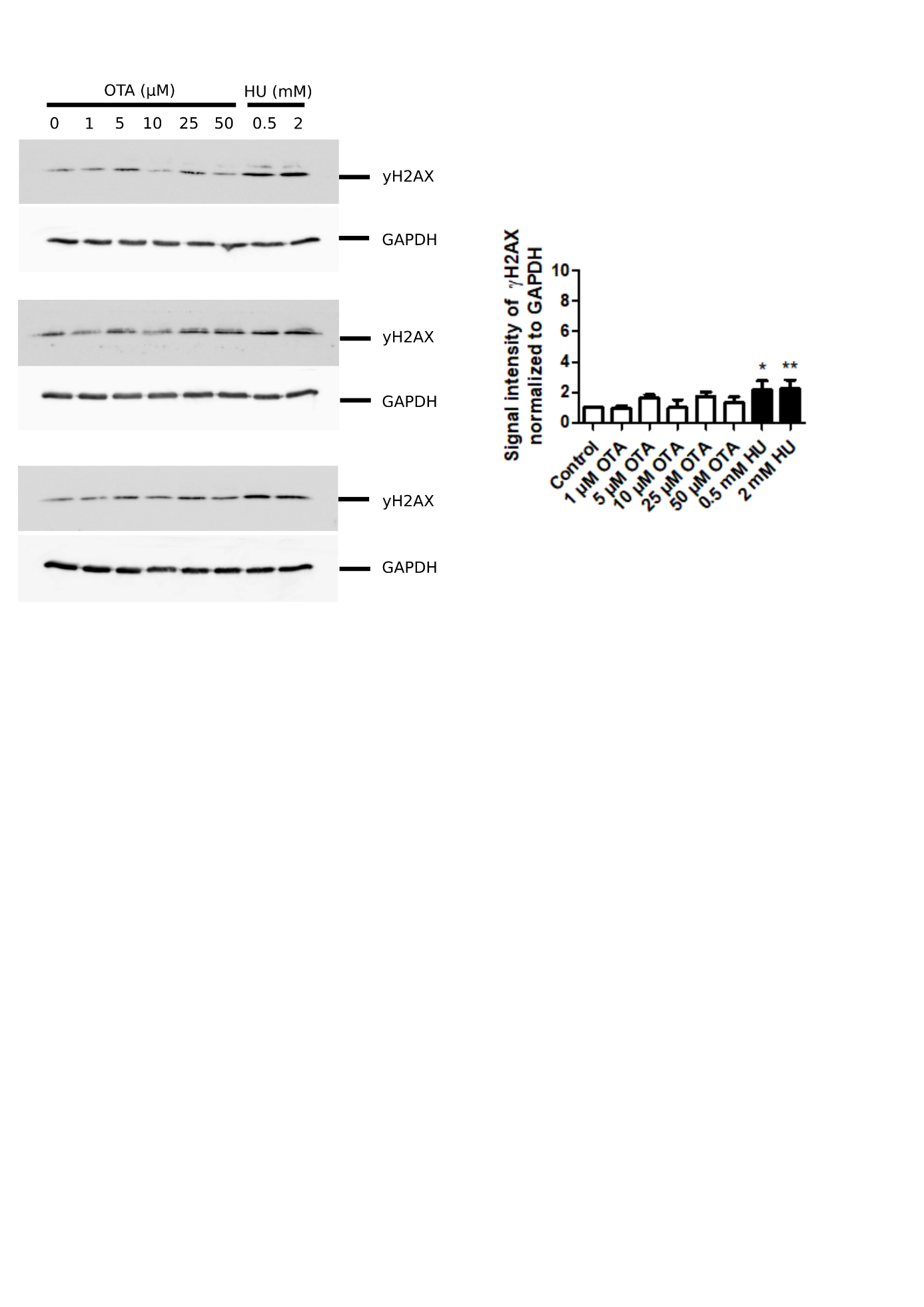
**

**Supplemental Figure 1. Western blot analysis of γH2AX** **in HK-2 cells exposed to OTA for 1 h.** Shown are western blot results of three independent experiments (n=3) and corresponding quantification. GAPDH served as loading control. Data are expressed as means ± SD relative to controls. Statistically significant changes compared to controls were determined by one-way ANOVA with Dunnett´s post-hoc test (*p≤0.05, **p≤0.01).

**Supplemental Figure 2**

**
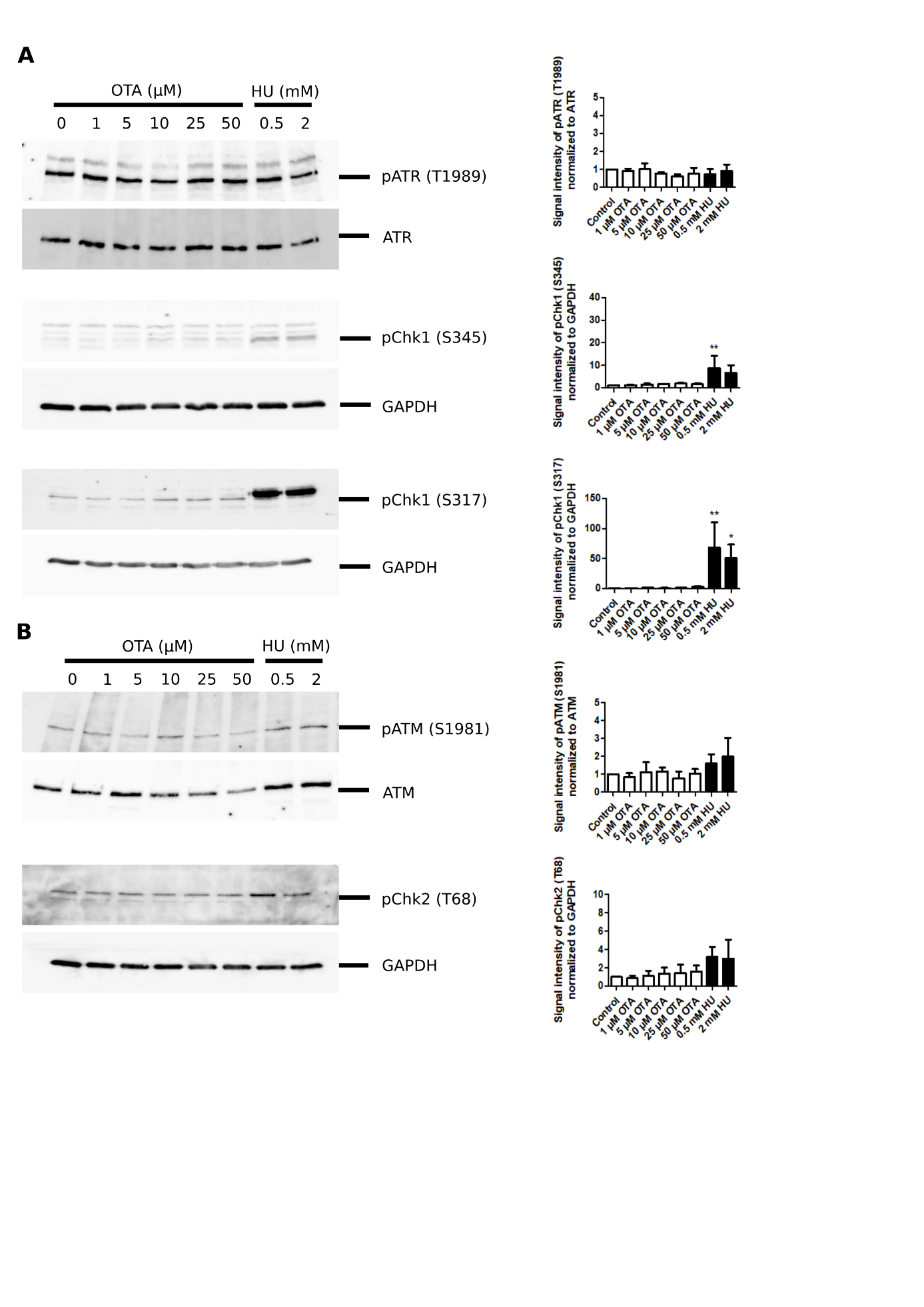
**

**Supplemental Figure 2. Analysis of ATR-Chk1 (A) and ATM-Chk2 (B) signaling pathways in HK-2 cells treated with OTA for 1 h.**

Western blot analysis of p-ATR (T1989), p-Chk1 (S345), p-Chk1 (S317), p-ATM (S1981) and p-Chk2 (T68) in HK-2 cells exposed to OTA (1 µM, 5 µM, 10 µM, 25 µM, 50 µM) or HU (0.5 mM, 2 mM) for 1 h. Western blot and corresponding quantification of western blot results by densitometry are representative of three independent experiments (n=3). ATR, ATM and GAPDH served as loading controls. Data are expressed as means ± SD relative to controls obtained from three biological replicates (n=3). Statistically significant changes compared to controls were determined by one-way ANOVA with Dunnett´s post-hoc test (*p≤0.05, **p≤0.01).

**Supplemental Figure 3**

**
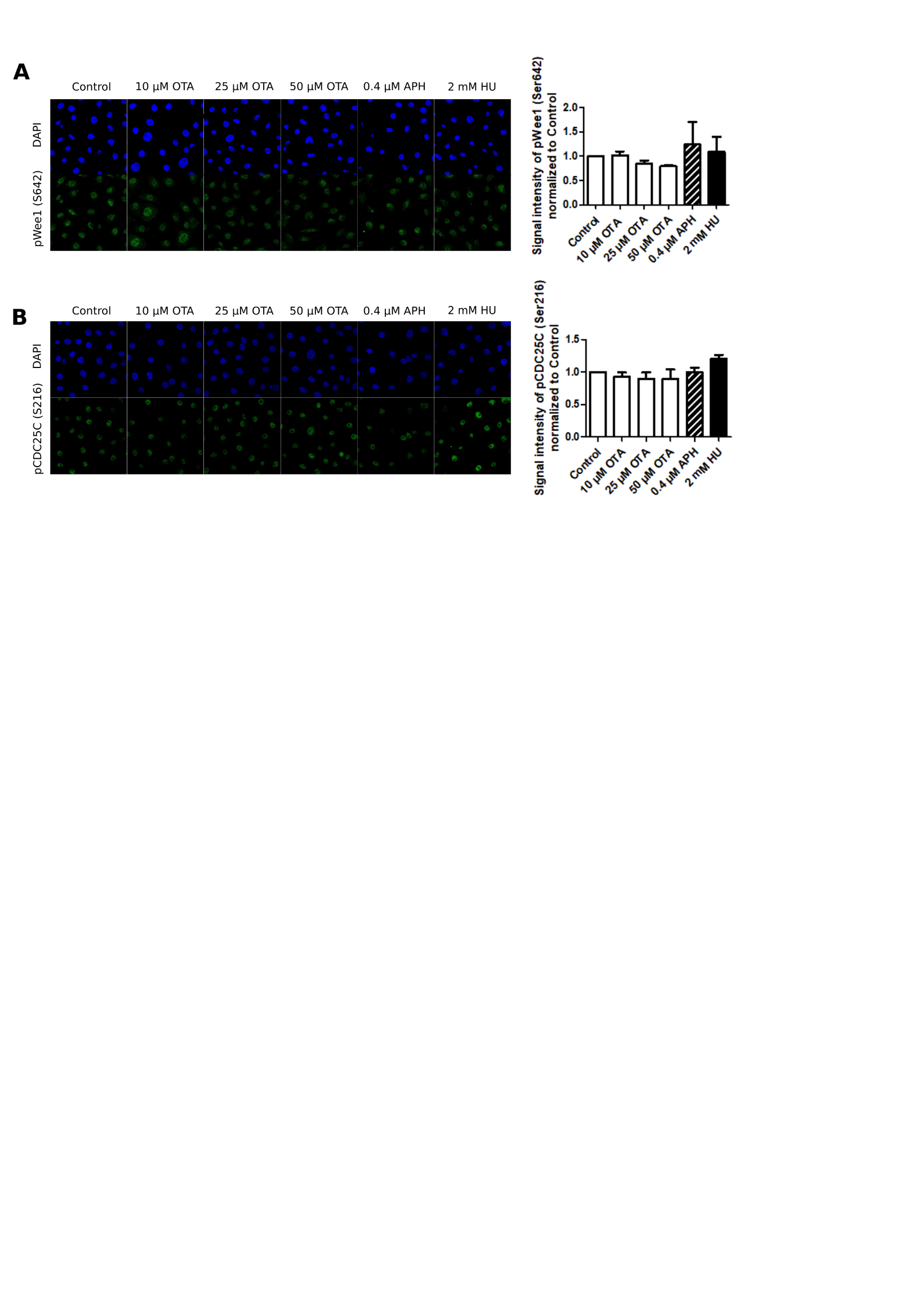
**

**Supplemental Figure 3. DNA damage effectors (Wee1, CDC25C) do not appear to be activated in response to OTA.**

**(A-B)** Immunofluorescence analysis of pWee1 (S642) and pCDC25C (S216) in HK-2 cells synchronized in late G1/S and treated with OTA (10 µM, 25 µM, 50 µM), APH (0.4 µM) and HU (2 mM) for 4 h during S phase. Immunofluorescence images and corresponding quantifications are representative of three biological experiments (n=3). Data are expressed as means ± SD relative to controls**.** Statistically significant changes compared to controls were determined by one-way ANOVA with Dunnett´s post-hoc test. Images were uniformly adjusted for brightness and contrast for better visualization.
